# Cavernous Sinus Cavernous Angioma Presenting As Painful Ophthalmoplegia: Report of a Rare Case Involving the Skull Base

**DOI:** 10.7759/cureus.106163

**Published:** 2026-03-30

**Authors:** Rodrigo Furlan Silva Fabri, Rodolfo Myronn De Melo Rodrigues, Anvitha Soundararajan

**Affiliations:** 1 Internal Medicine, Texas Tech University Health Sciences Center El Paso, El Paso, USA; 2 Internal medicine, Texas Tech University Health Sciences Center El Paso Paul L. Foster School of Medicine, El Paso, USA

**Keywords:** cavernous sinus cavernous angioma, cavernous sinus hemangioma, extra-axial vascular malformation, fronto-orbito-zygomatic approach, histopathologic confirmation, magnetic resonance imaging (mri), painful ophthalmoplegia, parasellar mass, skull base surgery, trigeminal neuralgia (ophthalmic division)

## Abstract

Cavernous sinus cavernous angiomas, also termed cavernous hemangiomas, are rare extra-axial vascular malformations with imaging and operative features that differ substantially from intraparenchymal cavernomas. Their presentation frequently mimics more common parasellar tumors, including meningiomas and schwannomas, rendering preoperative diagnosis difficult. These lesions are characteristically highly vascular, compressible, and prone to significant intraoperative hemorrhage.

We report a rare case of cavernous sinus cavernous angioma presenting with severe ophthalmic-division trigeminal neuralgia and complete ophthalmoplegia. The lesion was managed with surgical decompression and tumor debulking through a fronto-orbito-zygomatic approach. Clinical presentation, neuroimaging findings, intraoperative characteristics, histopathologic confirmation, and postoperative outcome are described in this report. A focused review of the literature highlights the diagnostic pitfalls, operative considerations, and contemporary management strategies for this uncommon skull base pathology.

## Introduction

Cavernous angiomas are low-flow vascular malformations composed of clusters of thin-walled sinusoidal vascular spaces lacking intervening neural tissue. While they are most frequently encountered as intraparenchymal lesions, extra-axial cavernous angiomas are uncommon, and involvement of the cavernous sinus represents only a small fraction of reported cases [1,2]. Overall, cavernous angiomas account for approximately 10% of intracranial vascular malformations and are identified in an estimated 0.4-0.8% of the general population [3]. Multiple lesions occur in up to 10% of patients, and approximately 5% demonstrate autosomal dominant inheritance associated with mutations in the *CCM1*, *CCM2*, or *CCM3 *genes [3].

The cavernous sinus is a complex venous structure containing cranial nerves III, IV, the ophthalmic (V1) and maxillary (V2) divisions of the trigeminal nerve, cranial nerve VI, and the cavernous segment of the internal carotid artery. Consequently, lesions arising in this region frequently manifest with neuro-ophthalmologic symptoms, including trigeminal neuralgia, facial hypoesthesia, diplopia, ptosis, ophthalmoplegia, retro-orbital pain, and visual impairment [1,2,4,5]. Painful ophthalmoplegia is a particularly characteristic presentation and often prompts radiologic evaluation.

Radiographically, cavernous sinus cavernous angiomas differ from parenchymal cavernomas in that they lack the classic “popcorn” or “mulberry” appearance and hemosiderin rim. Instead, they typically present as well-circumscribed parasellar masses that are markedly hyperintense on T2-weighted magnetic resonance imaging and demonstrate strong, often homogeneous, contrast enhancement [4-6]. These features overlap substantially with those of parasellar meningiomas and schwannomas, leading to frequent misdiagnosis before surgery [1,4,6]. Even with advanced imaging techniques, definitive diagnosis is often established only intraoperatively.

Surgical management of cavernous sinus cavernous angiomas is technically demanding because of their extreme vascularity, compressibility, and close relationship with critical neurovascular structures. Historically, attempts at aggressive resection were associated with high rates of intraoperative hemorrhage and neurological morbidity [1,2]. Advances in skull base surgery, microsurgical techniques, and operative planning, particularly the use of the fronto-orbito-zygomatic approach, have improved exposure and enabled safer internal debulking and decompression while minimizing cranial nerve injury [7-9]. In parallel, stereotactic radiosurgery has emerged as an effective alternative or adjunct treatment, particularly for large or residual lesions, with favorable long-term tumor control and symptom improvement reported in recent series and meta-analyses. In selected cases, endoscopic endonasal approaches have also been explored as an alternative surgical route for cavernous sinus lesions, including cavernous sinus cavernous angiomas. These approaches may offer a direct midline corridor with reduced brain retraction in carefully chosen patients; however, their applicability remains limited by tumor size, lateral extension, vascularity, and close relationships with critical neurovascular structures. Consequently, open skull base approaches remain the most commonly employed strategies for symptomatic or laterally extensive lesions [10-15].

## Case presentation

A 62-year-old woman presented with a four-week history of severe left-sided retro-orbital pain radiating along the ophthalmic division (V1) of the trigeminal nerve. Her symptoms were progressive and accompanied by diplopia that ultimately evolved into complete left ophthalmoplegia. She denied trauma, constitutional symptoms, or a prior history of malignancy.

**Figure 1:**
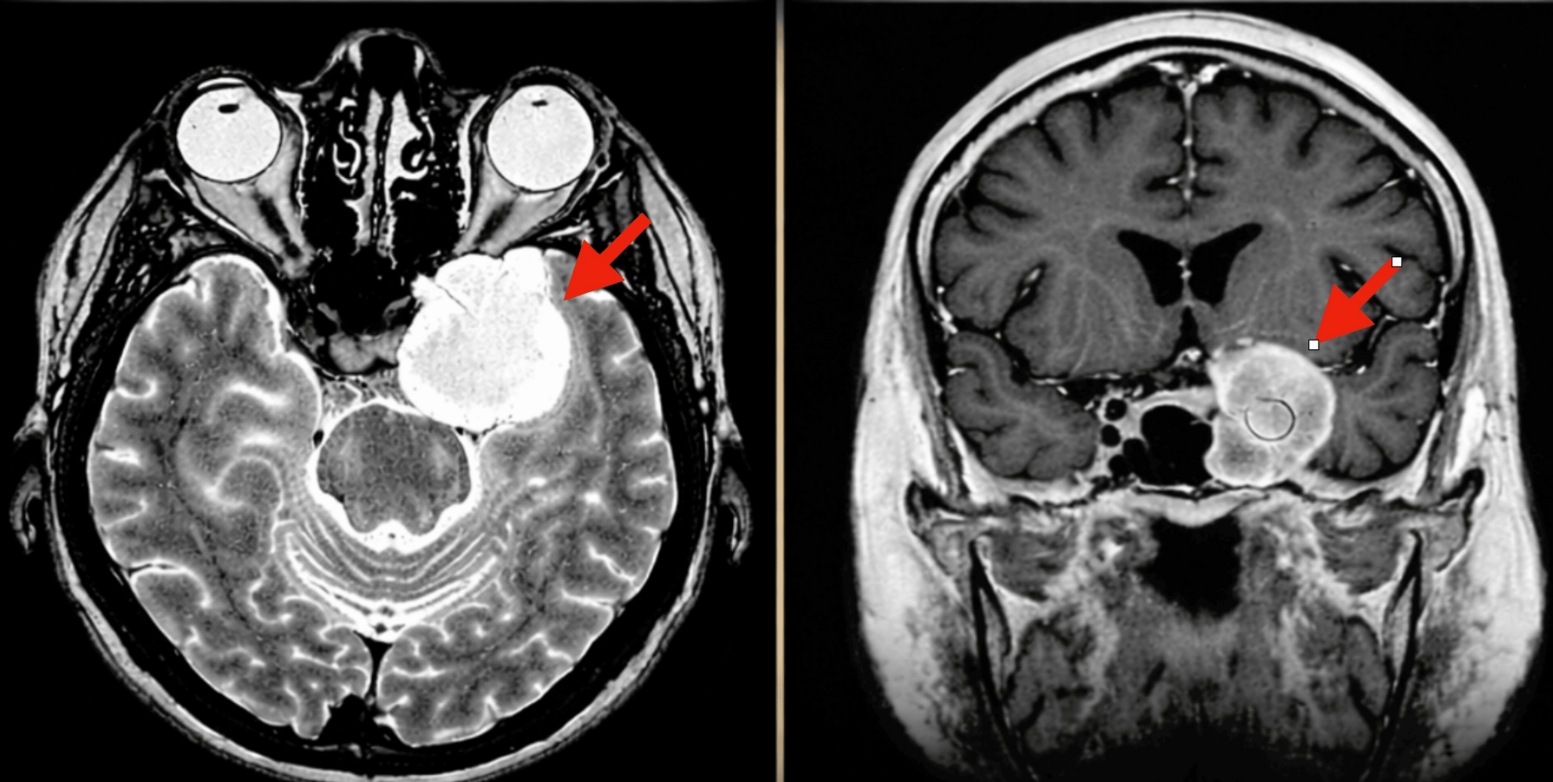
Axial and Coronal MRI Demonstrating a Left Cavernous Sinus Cavernous Angioma Axial T2-weighted (left) and coronal T1-weighted post-contrast (right) MRI images revealing a well-defined, expansile lesion centered within the left cavernous sinus. The mass demonstrates smooth contour expansion with displacement of adjacent parasellar structures, characteristic of a cavernous sinus cavernous angioma (red arrows).

Magnetic resonance imaging (MRI) of the brain demonstrated a well-circumscribed expansile lesion completely occupying the left cavernous sinus. The mass was markedly hyperintense on T2-weighted sequences and demonstrated strong homogeneous enhancement on post-contrast T1-weighted imaging. Smooth enlargement of the cavernous sinus with medial displacement of the cavernous segment of the internal carotid artery was noted. Gradient-echo sequences demonstrated subtle blooming suggestive of blood products, although not definitive. The radiologic features-particularly the intense T2 hyperintensity, homogeneous enhancement, smooth cavernous sinus expansion, and absence of a dural tail-favored the diagnosis of a cavernous sinus cavernous angioma rather than a parasellar meningioma or schwannoma (Figure 1).

**Figure 2:**
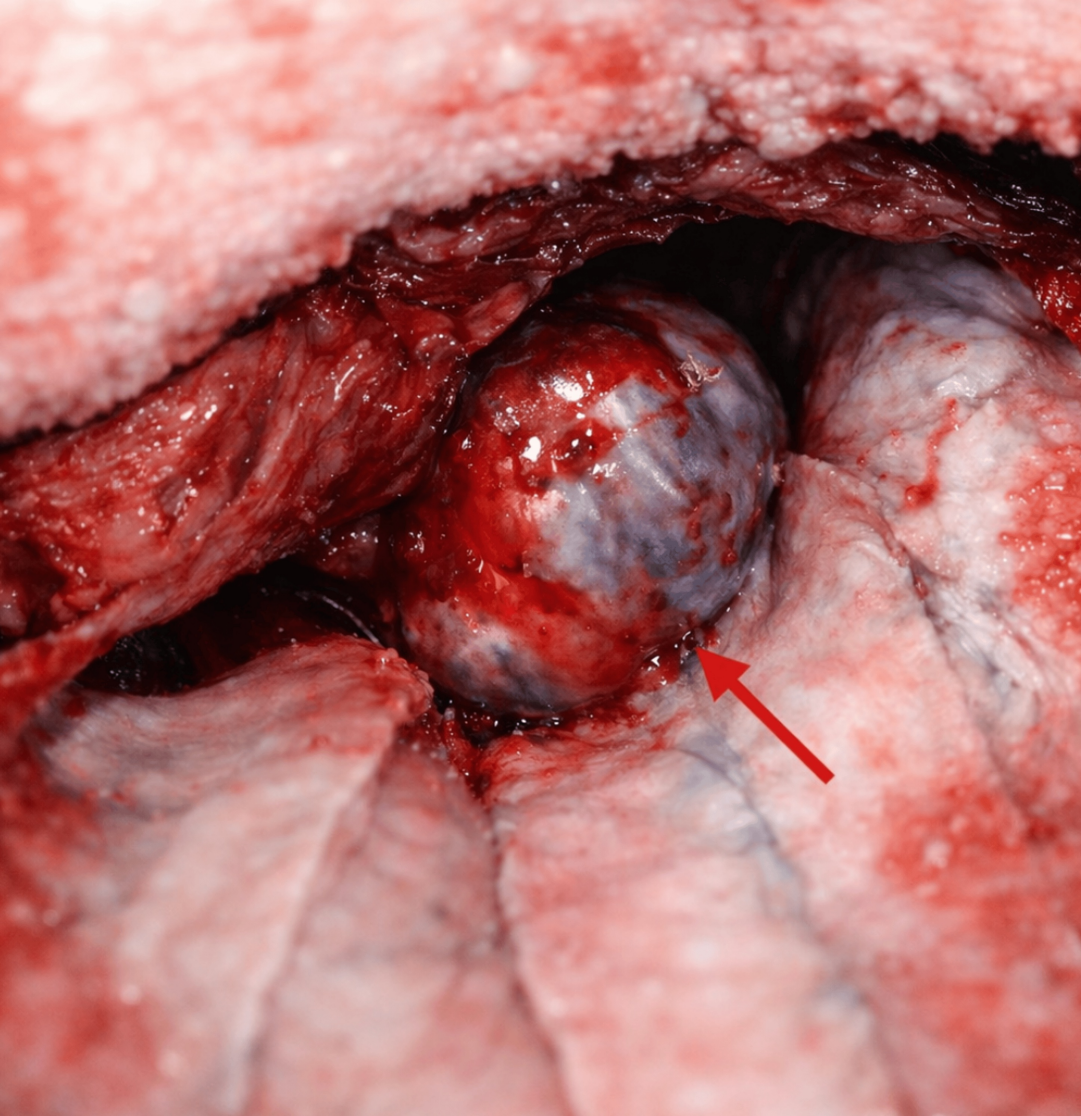
Intraoperative View Demonstrating Cavernous Sinus Cavernous Angioma Intraoperative photograph obtained through a fronto-orbito-zygomatic approach. The red arrow points to a bluish, highly vascular, compressible mass arising from the cavernous sinus. The lesion expands outward without a clear capsule and demonstrates the characteristic soft, venous appearance typical of a cavernous sinus cavernous angioma.

Given the patient’s progressive cranial neuropathies and radiologic findings, surgical intervention was pursued. A left fronto-orbito-zygomatic craniotomy was performed to provide wide exposure of the cavernous sinus and adjacent skull base structures. The anterior clinoid process was not removed, as adequate exposure of the cavernous sinus was achieved through the fronto-orbito-zygomatic approach. Intraoperatively, the lesion appeared as a dark-bluish, highly vascular, compressible mass arising from within the cavernous sinus (Figure 2). The tumor demonstrated slow re-expansion following bipolar coagulation, consistent with a venous vascular lesion. No clear dissection plane was identified between the lesion and cranial nerves III, IV, V1, or VI, and the lateral wall of the cavernous sinus could not be safely mobilized. Internal debulking was performed using bipolar coagulation and gentle suction. Hemostasis required extensive application of hemostatic agents due to diffuse venous oozing. The A maximal safe resection strategywas adopted, prioritizing internal decompression while avoiding aggressive dissection from cranial nerves in the absence of a clear surgical plane.

**Figure 3:**
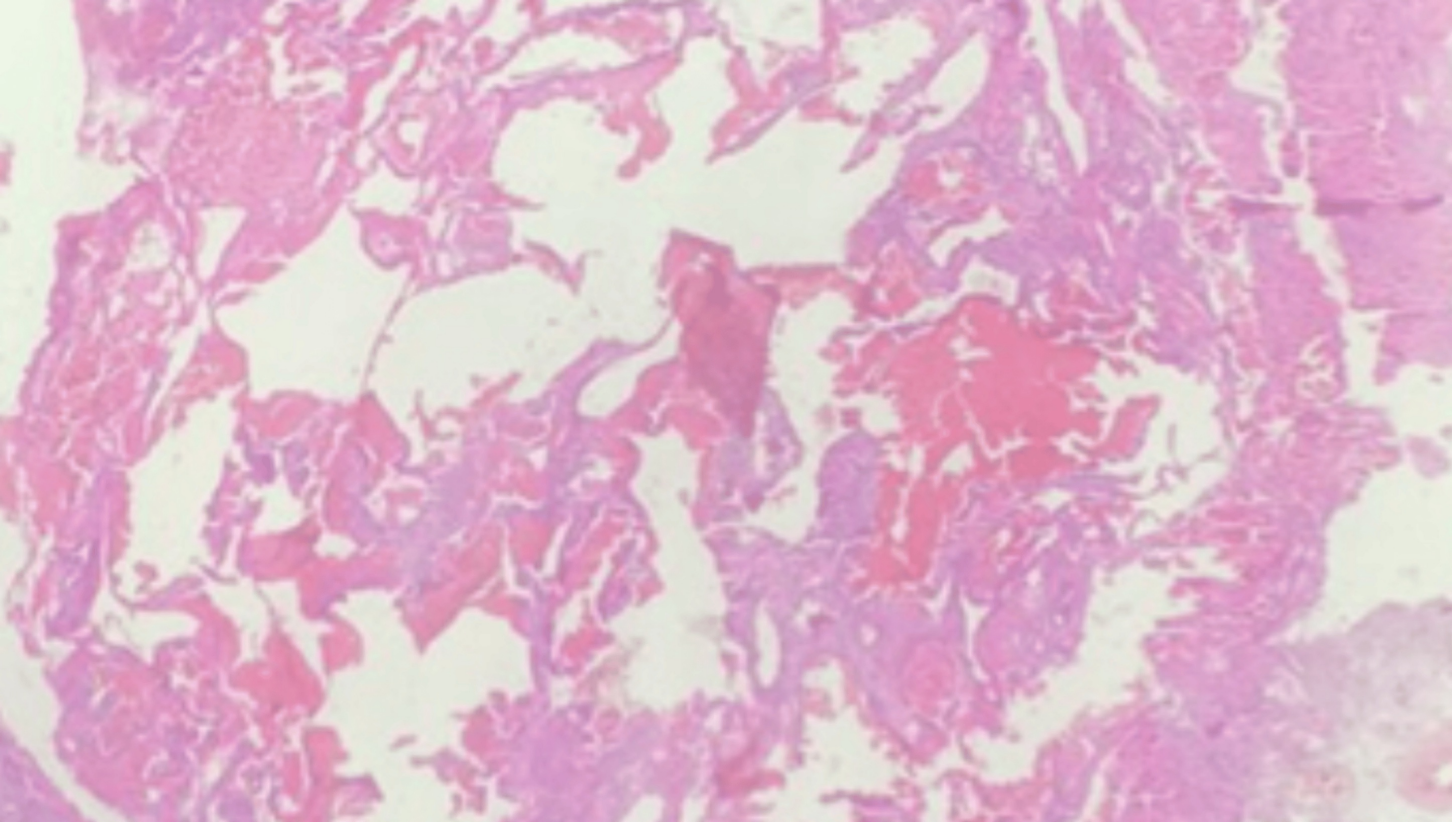
Histopathology of Cavernous Sinus Cavernous Angioma Histopathology of cavernous sinus cavernous angioma. Hematoxylin and eosin staining demonstrates multiple dilated, thin-walled sinusoidal vascular spaces lined by flattened endothelial cells, consistent with cavernous angioma.

Histopathologic examination of the resected specimen demonstrated multiple dilated, thin-walled sinusoidal vascular channels lined by flattened endothelial cells and lacking intervening neural tissue. The vascular spaces were separated by fibrous connective tissue stroma without cytologic atypia or mitotic activity. These findings are consistent with cavernous angioma (cavernous hemangioma) (Figure 3).

**Figure 4:**
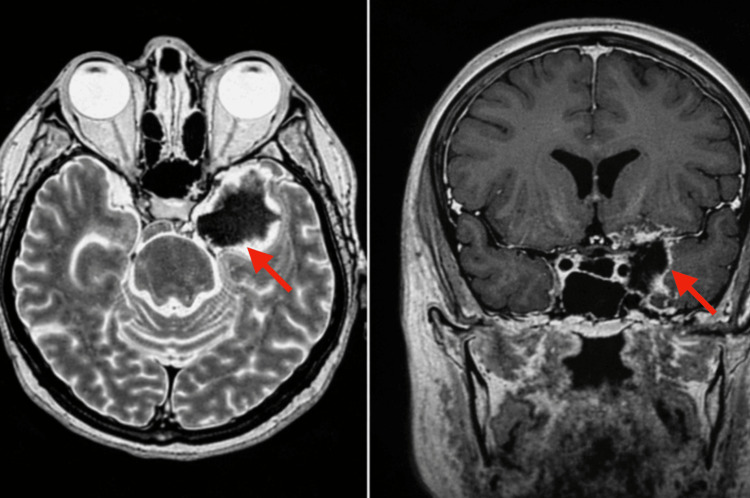
Postoperative Follow-up MRI Demonstrating Debulking of a Left Cavernous Sinus Lesion Axial T2-weighted MRI (left) and coronal T1-weighted post-contrast MRI (right) obtained during postoperative follow-up demonstrate interval debulking of the previously identified lesion in the left cavernous sinus. Imaging shows postoperative changes with reduction of the mass effect and no radiographic evidence of residual tumor (red arrows).

The patient’s postoperative course was uneventful. She experienced early improvement in trigeminal pain and gradual recovery of partial ocular motility over six weeks. A follow-up MRI performed approximately four weeks after surgery demonstrated postoperative changes with reduction of mass effect and no radiographic evidence of residual tumor, consistent with satisfactory tumor debulking (Figure 4).

The patient is currently undergoing serial MRI surveillance to monitor for recurrence. At present, given the absence of radiographic residual disease and clinical improvement, adjuvant stereotactic radiosurgery is not indicated; however, it remains a potential option in the event of future recurrence or progression.

## Discussion

Cavernous sinus cavernous hemangiomas are rare, extra-axial vascular tumors that account for a small fraction of benign cavernous sinus lesions and are characterized by thin-walled, sinusoidal vascular spaces with slow flow [1-3]. Clinically, they often present with progressive cranial neuropathies due to mass effect on the oculomotor, trochlear, trigeminal, and abducens nerves, with diplopia, ophthalmoplegia, and trigeminal pain being among the most frequent symptoms [1-3]. The painful, rapidly progressive ophthalmoplegia in our patient is in line with the typical pattern reported in surgical and radiologic series of cavernous sinus hemangiomas [1,2].

Radiologically, these lesions are notorious for mimicking cavernous sinus meningiomas and schwannomas. Characteristic MRI features include marked T2 hyperintensity, strong and often homogeneous contrast enhancement, smooth cavernous sinus expansion, and displacement rather than encasement of the internal carotid artery [4-6]. Montoya et al. emphasized that very high T2 signal and specific enhancement patterns can strongly suggest cavernous sinus hemangioma, yet preoperative misdiagnosis remains common [4]. In the systematic review by Osunronbi et al. (338 reported cases), the majority of lesions were initially thought to be meningiomas or other parasellar tumors, underscoring the diagnostic challenge [6]. In our case, the homogeneous parasellar mass without calcification or dural tail raised suspicion for a vascular lesion, but definitive diagnosis was ultimately based on intraoperative findings.

Historically, open microsurgical resection was the primary treatment modality. Early series by Shi et al. and Zhou et al. demonstrated that surgical removal can decompress cranial nerves and achieve good long-term control, but at the cost of considerable intraoperative bleeding and nontrivial morbidity [1,3]. Linskey and Sekhar highlighted that these tumors may bleed profusely and frequently lack a clear dissection plane from adjacent neurovascular structures, making aggressive resection hazardous [2]. More contemporary surgical series from high-volume skull base centers have refined technique and selection. Large cohorts from Nguyen et al., Li et al., and Goel et al. reported that microsurgical debulking via skull base approaches (including fronto-orbito-zygomatic and extended frontotemporal routes) can be performed with improved safety, yet cranial nerve deficits and significant blood loss remain important concerns, particularly for large or giant lesions [7-9].

Our experience aligns with this modern “maximal safe resection” philosophy. Using a fronto-orbito-zygomatic approach, we prioritized internal debulking and decompression of the cavernous sinus while intentionally avoiding forceful dissection from cranial nerves when no clear plane was present. This strategy mirrors the approach advocated in contemporary surgical series, where subtotal removal is often accepted to reduce the risk of permanent neurologic morbidity [7-9]. The patient’s postoperative improvement in trigeminal pain and gradual recovery of ocular motility are consistent with the favorable functional outcomes reported after decompressive surgery [1,3,7]. The fronto-orbito-zygomatic approach was selected to provide wide extradural and intradural exposure of the cavernous sinus, allowing early identification of neurovascular structures and controlled internal decompression of the lesion. Given the absence of a clear dissection plane between the tumor and cranial nerves, a maximal safe resection strategy was prioritized over aggressive gross total excision to minimize neurological morbidity.

Over the past two decades, stereotactic radiosurgery (SRS), particularly Gamma Knife® radiosurgery (GKRS; Elekta AB, Stockholm, Sweden), has emerged as a highly effective and less invasive treatment option for cavernous sinus hemangiomas. Early radiosurgical reports and a meta-analysis by Wang et al. showed high rates of tumor volume reduction (>90%) with low rates of new cranial neuropathies and durable tumor control [10,11]. Subsequent single- and multicenter studies have consistently demonstrated excellent radiographic and clinical outcomes. Lee et al. reported long-term control with symptomatic improvement in most patients treated primarily with GKRS [12]. Park et al. described favorable long-term clinical outcomes and proposed that GKRS may be considered first-line therapy in patients at high surgical risk [10]. More recently, Yang and colleagues reported outstanding results with staged GKRS for giant cavernous sinus hemangiomas and with personalized, volume-adapted GKRS in a cohort of 187 patients, achieving tumor control rates close to 100% with substantial shrinkage and low morbidity [13,14]. Temporal volumetric analyses, such as those by Cho et al., further confirm progressive and sustained tumor shrinkage after radiosurgery in the majority of cases [15].

The pooled analysis by Osunronbi et al. provides a comprehensive view of modern practice: radiosurgery alone or as part of multimodality management is associated with high rates of symptom improvement and fewer complications compared with surgery alone, especially for larger or surgically inaccessible lesions [6]. In this context, our case illustrates the role of microsurgery in carefully selected patients: surgical debulking remains valuable when a lesion is highly symptomatic, when rapid decompression of cranial nerves is required, or when diagnostic uncertainty persists despite imaging. At the same time, the growing body of evidence suggests that radiosurgery - either as primary therapy or as an adjuvant for residual tumor - should be strongly considered, particularly for large or giant cavernous sinus hemangiomas and in patients for whom the risk of open surgery is unacceptably high [6,10-15].

Taken together, current data support an individualized, multimodal approach. For our patient, open microsurgical debulking via a fronto-orbito-zygomatic route provided immediate decompression and symptomatic relief. Based on the contemporary literature, adjuvant GKRS would be a reasonable option if a clinically significant residual or recurrent tumor is identified on follow-up imaging [6,10-15].

## Conclusions

Cavernous sinus cavernous angiomas are rare vascular lesions that can closely mimic more common parasellar tumors and frequently present with painful ophthalmoplegia due to involvement of cranial nerves within the cavernous sinus. Although MRI may provide important diagnostic clues, definitive diagnosis is often established intraoperatively due to the lesion’s characteristic vascularity and lack of a clear dissection plane. Surgical management remains challenging given the complex cavernous sinus anatomy and the proximity of critical neurovascular structures. A maximal safe resection strategy, supported by careful preoperative planning and meticulous microsurgical technique, is essential to achieve effective decompression while minimizing neurological morbidity. Awareness of this entity is critical for accurate diagnosis, appropriate surgical planning, and favorable clinical outcomes.
